# Supplementary material for: PTEN loss in glioma cell lines leads to increased extracellular vesicle biogenesis and PD-L1 cargo in a PI3K-dependent manner
Source: J Biol Chem. 2024 Dec 26;301(2):108143. doi: 10.1016/j.jbc.2024.108143 (PMC11791317; doi:10.1016/j.jbc.2024.108143)
Supplement: Supporting Information [file mmc1.docx]

**A B C**

**
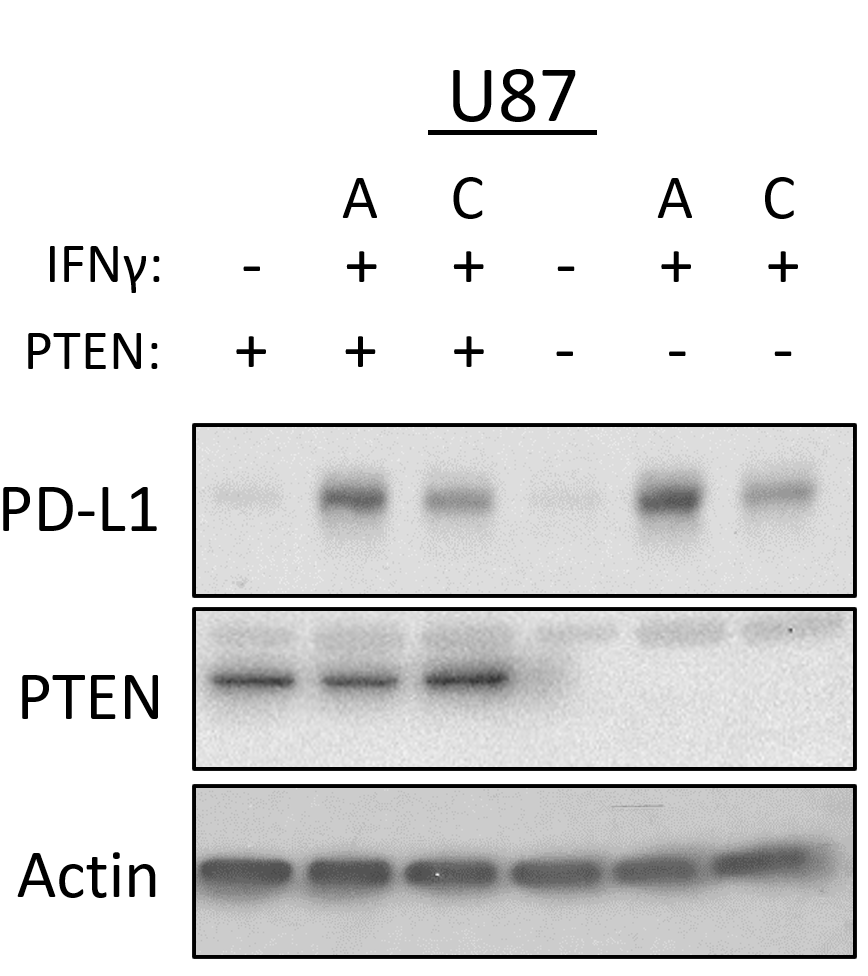


**
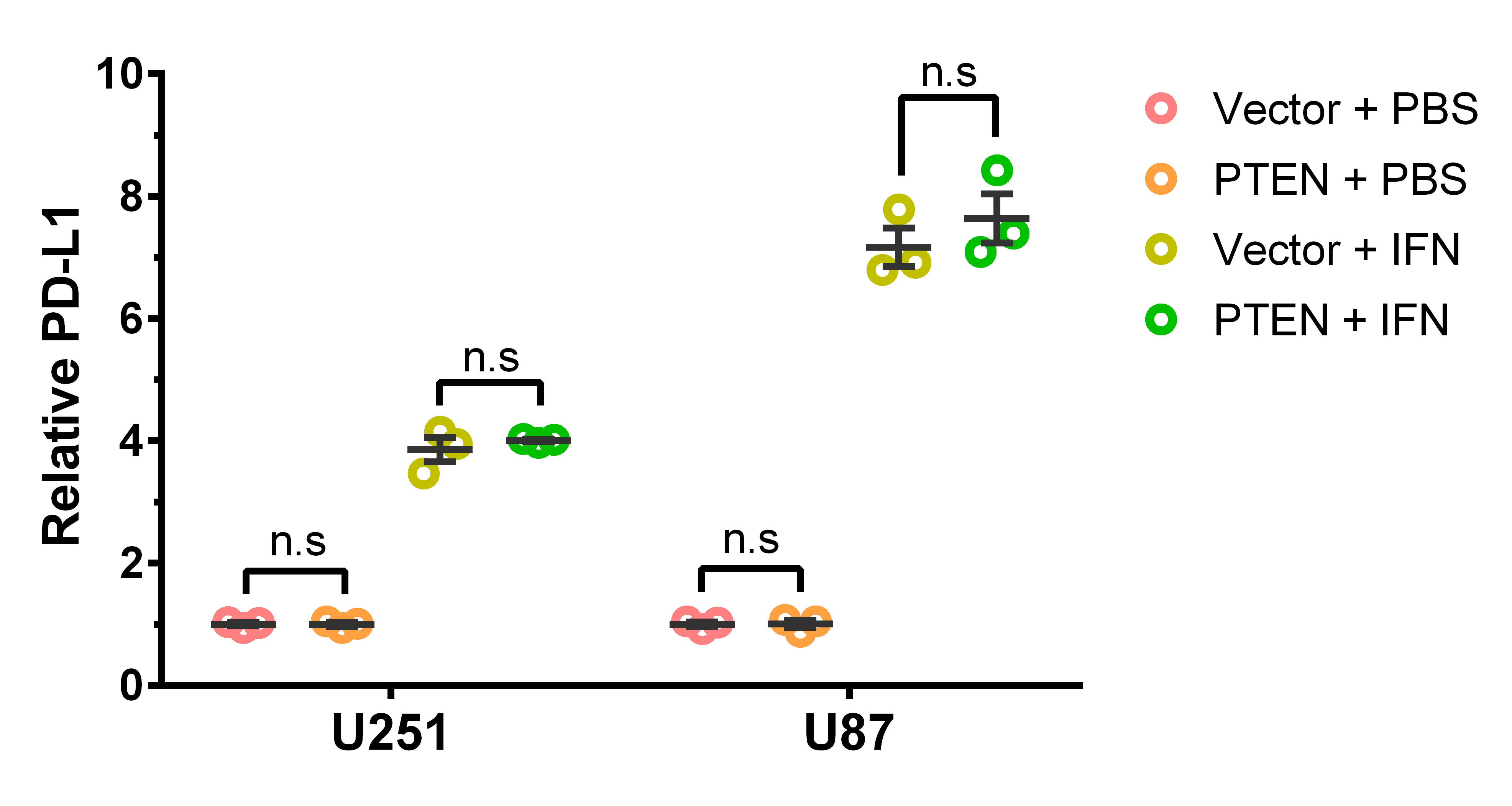


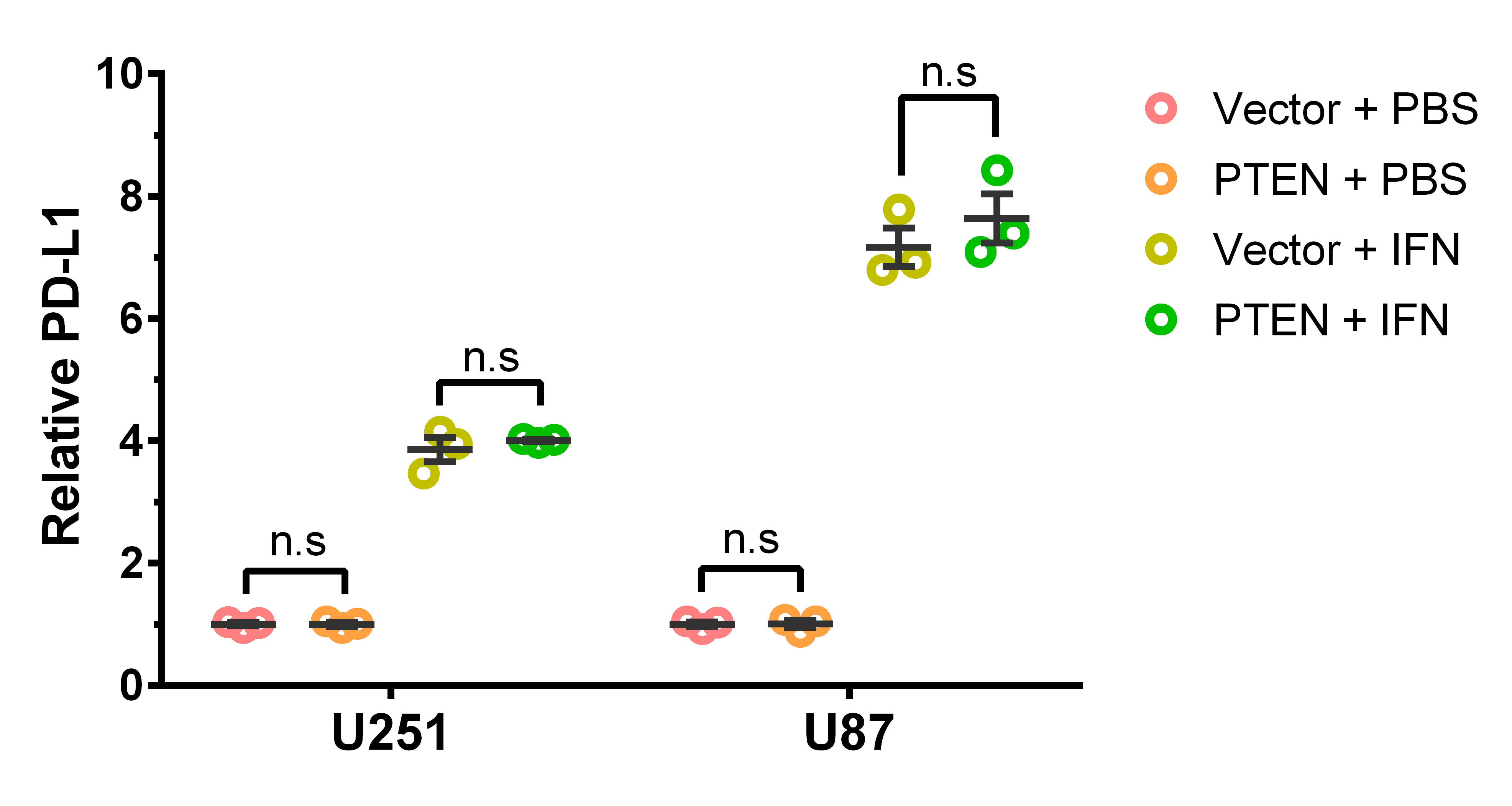


**D E F**

**
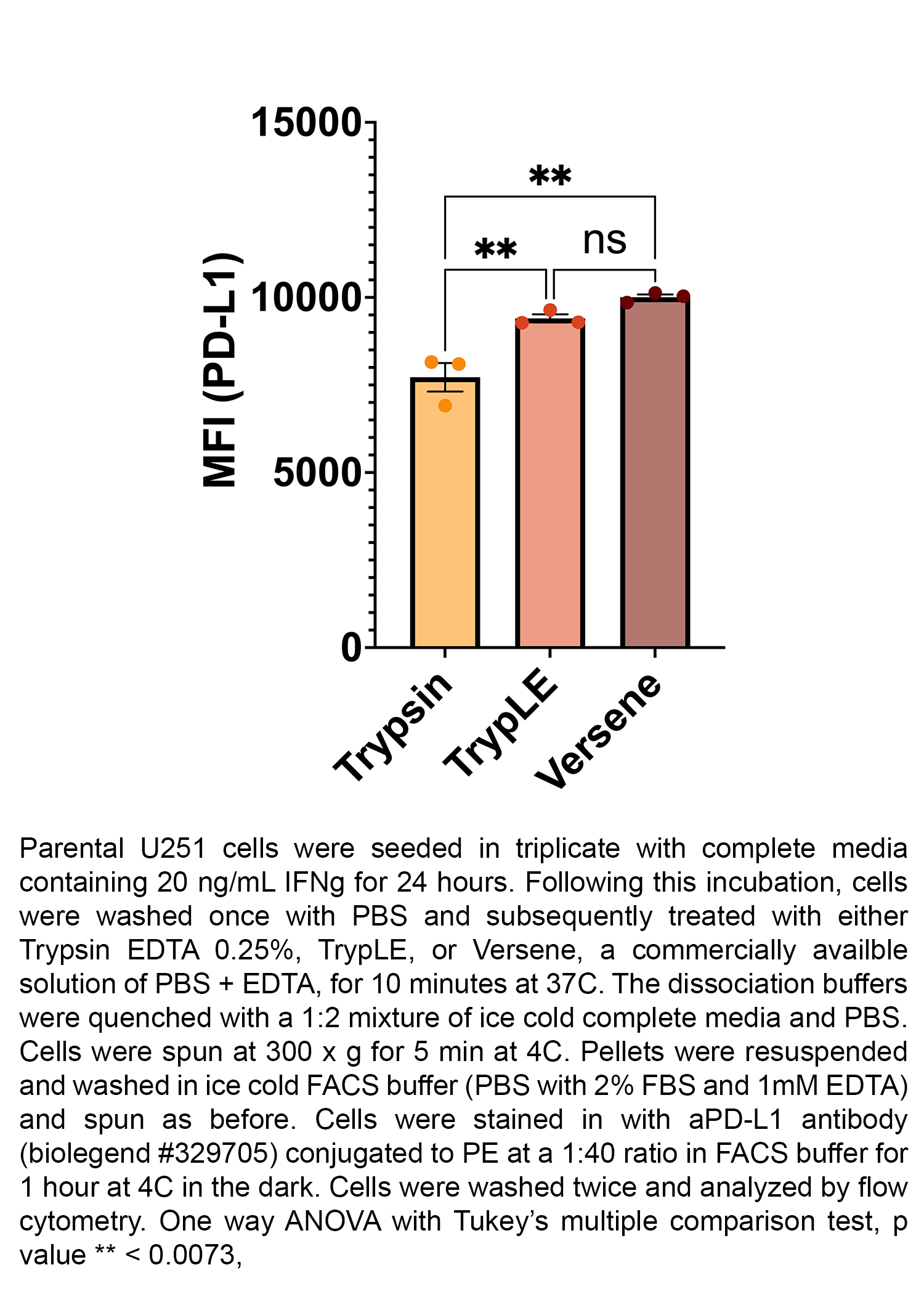

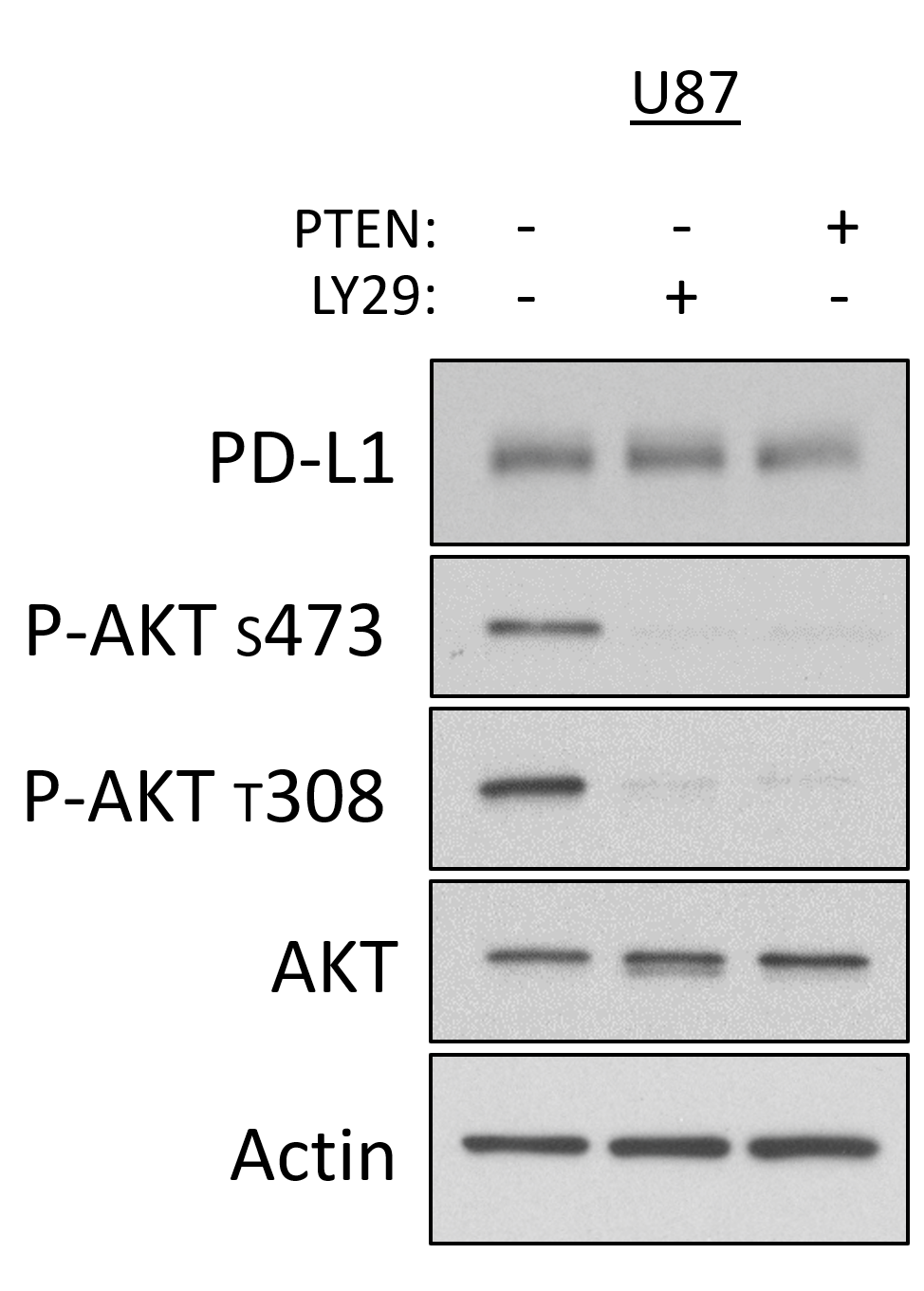
**

**
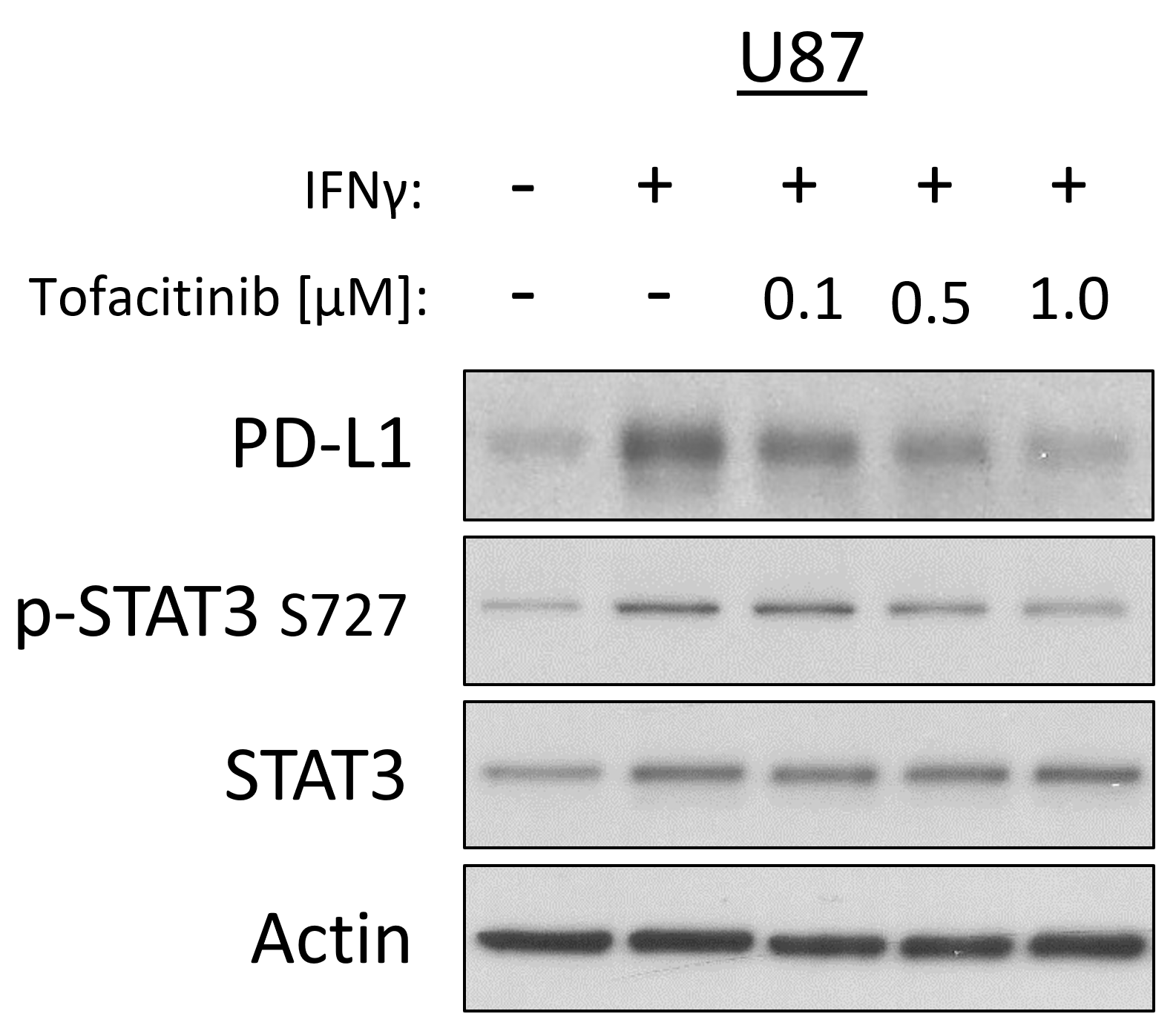
**

**Supporting Figure 1. IFN-γ upregulates PD-L1 in a PI3K/PTEN-independent manner**

**A.)** Semi-stable isogenic U87 and U251 cells expressing either the control empty vector or PTEN vector were treated once with IFN-γ (50 ng/mL) or PBS for 24 hours. RNA was isolated from each treatment group and subjected to reverse transcription (RT) PCR to generate cDNA and used to detect PD-L1 mRNA transcripts by qPCR. Within each cell type, actin served as a housekeeping control gene to calculate the relative quantity of PD-L1 transcripts for each condition using the 2^-ΔΔCt^ method. **B.)** PD-L1 expression from all whole cell lysates across all immunoblot experiments were quantified by densitometry (mean gray value) and normalized based on actin. Paired T test, p-value = 0.64. **C.)** Semi-stable U87 cells expressing either the control empty vector or the PTEN vector were either treated once with IFN-γ (50 ng/mL) for 15 hours, denoted with “A” for acute response, or cultured with media supplemented with IFN-γ for five passages to represent a chronic state of inflammatory signaling and is denoted as “C.” Immunoblot analysis was performed to compare PD-L1 and PTEN expression with actin serving as a loading control. **D.)** Two groups of U87 cells were transiently transduced with an empty vector (-) control and one group with the PTEN vector (+). Two days later, one empty vector control group was treated with the PI3K inhibitor LY294002 (LY29, 30 μM), the other two groups were treated with DMSO as the vehicle control. All groups were concomitantly treated with IFN-γ (50 ng/mL). Twenty-four hours after treatment, cells were lysed and immunoblot analysis was performed to compare PD-L1, PTEN, total AKT, AKT activity, (T308, S473), and actin as the loading control. **E.)** In a similar fashion, U87 cells were concomitantly treated with the JAK/STAT inhibitor, Tofacitinib (CP-690550 citrate) or with DMSO (-) as the vehicle control, and IFN-γ (50 ng/mL). Immunoblot analysis was performed 16-hours post-treatment to compare PD-L1 expression and STAT3 activity (S727), with actin serving as the loading control. **F)** Parental U251 cells were seeded in triplicate with complete media containing 20 ng/mL IFN-γ for 24 hours. Following this incubation, cells were washed once with PBS and subsequently treated with either Trypsin EDTA 0.25%, TrypLE, or Versene, a commercially available solution of PBS + EDTA, for 10 minutes at 37C. Cells were washed in ice cold PBS, centrifuged, resuspended in FACS buffer and analyzed by flow cytometry. One way ANOVA with Tukey’s multiple comparison test, p value ** < 0.0073.

**A**

**
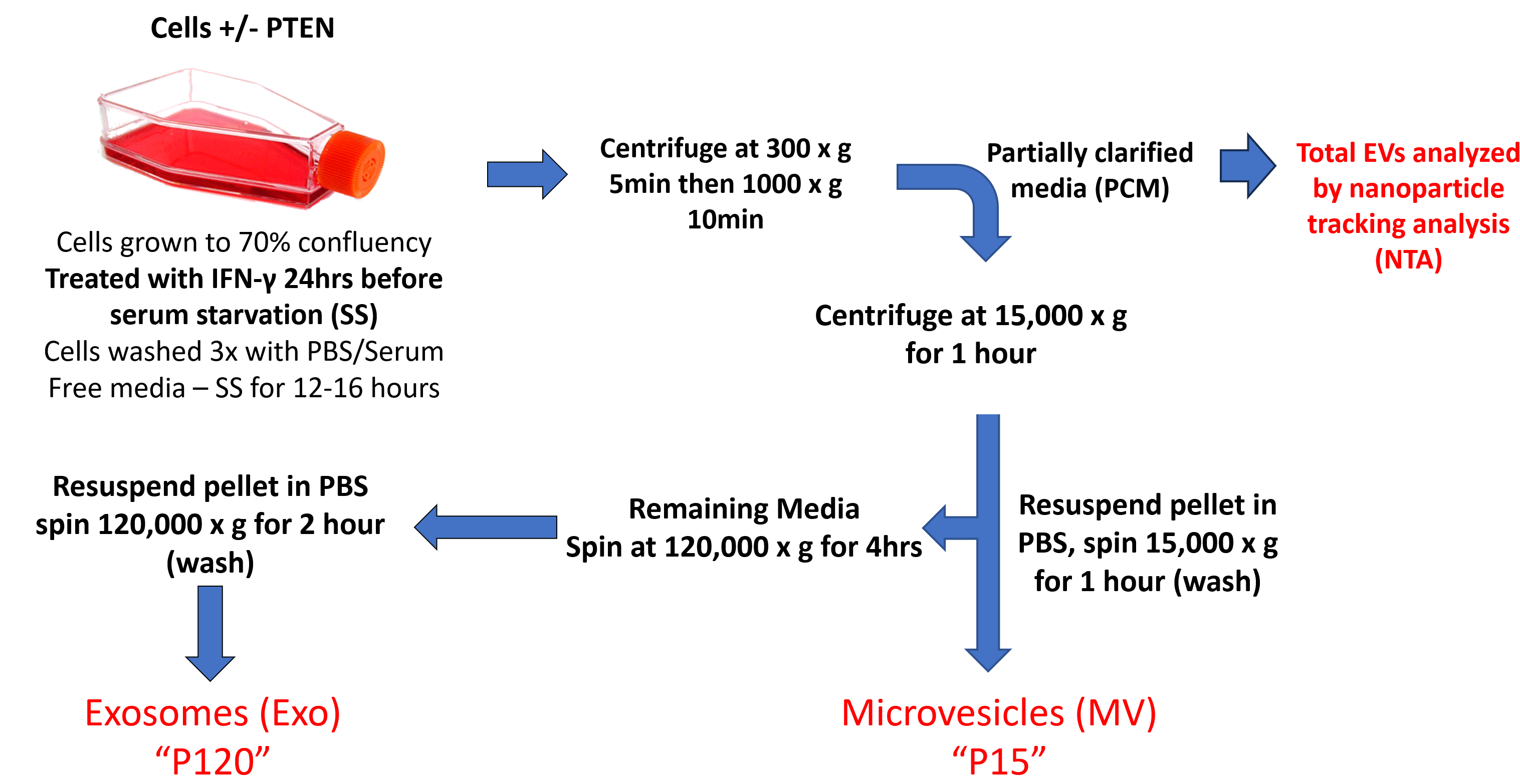
**

**B C D**

**
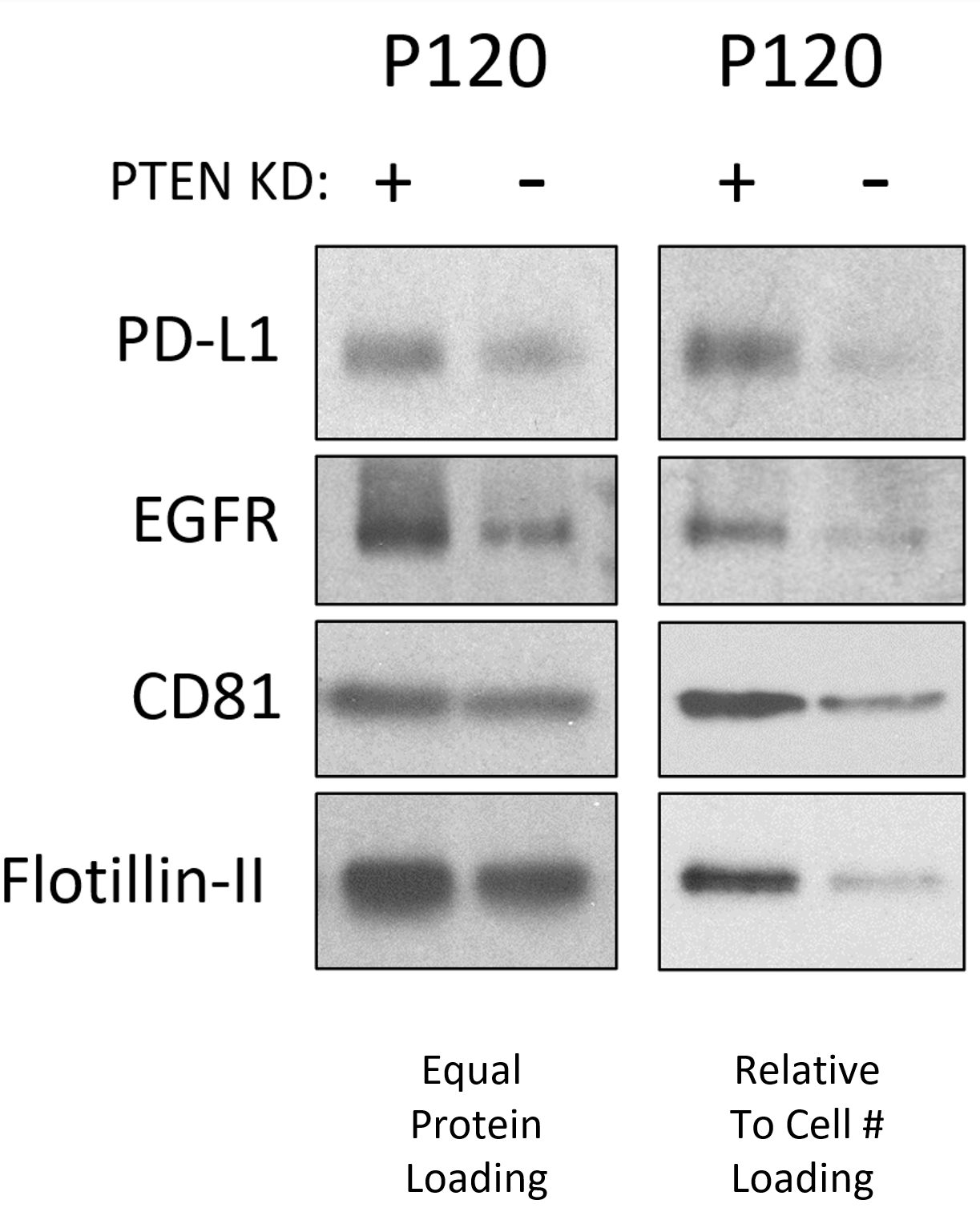

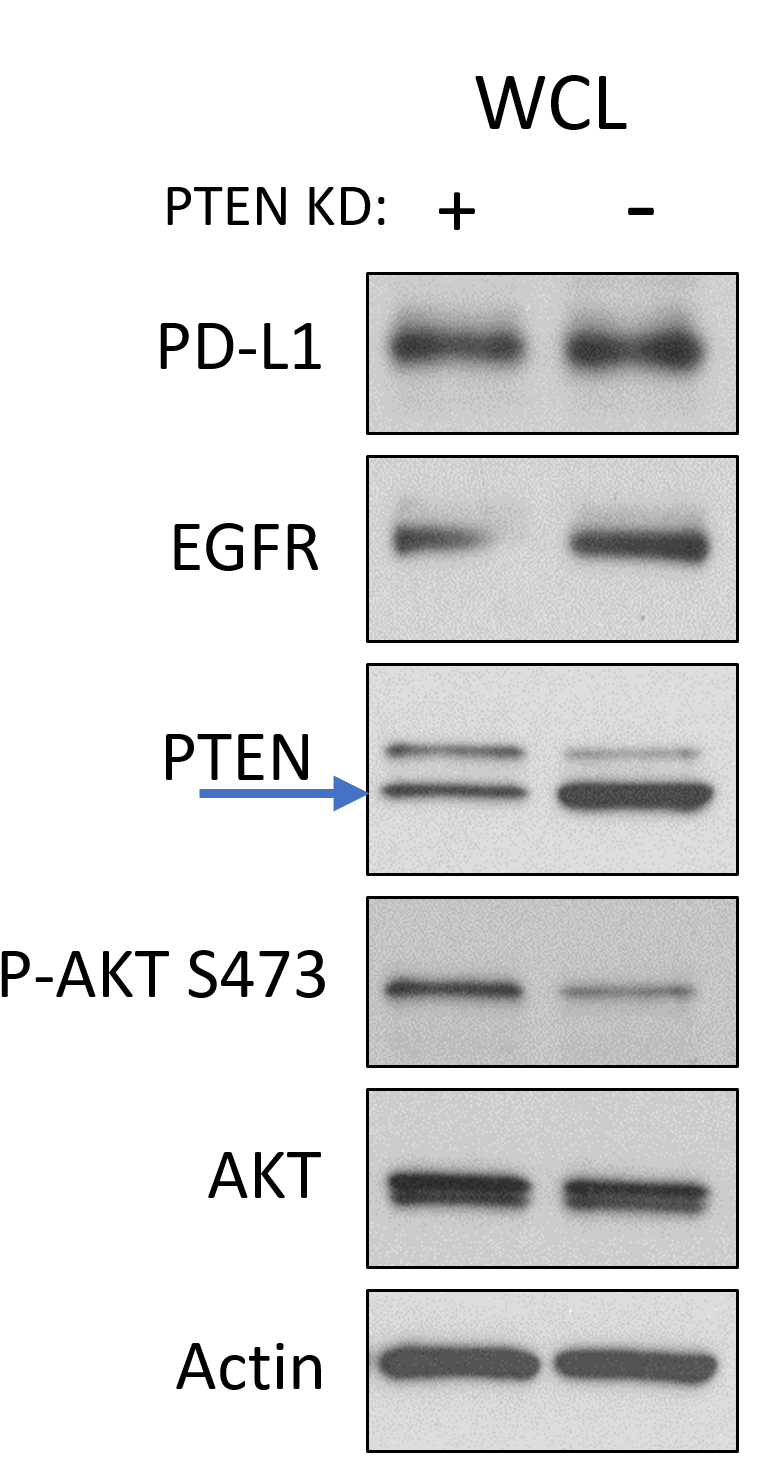

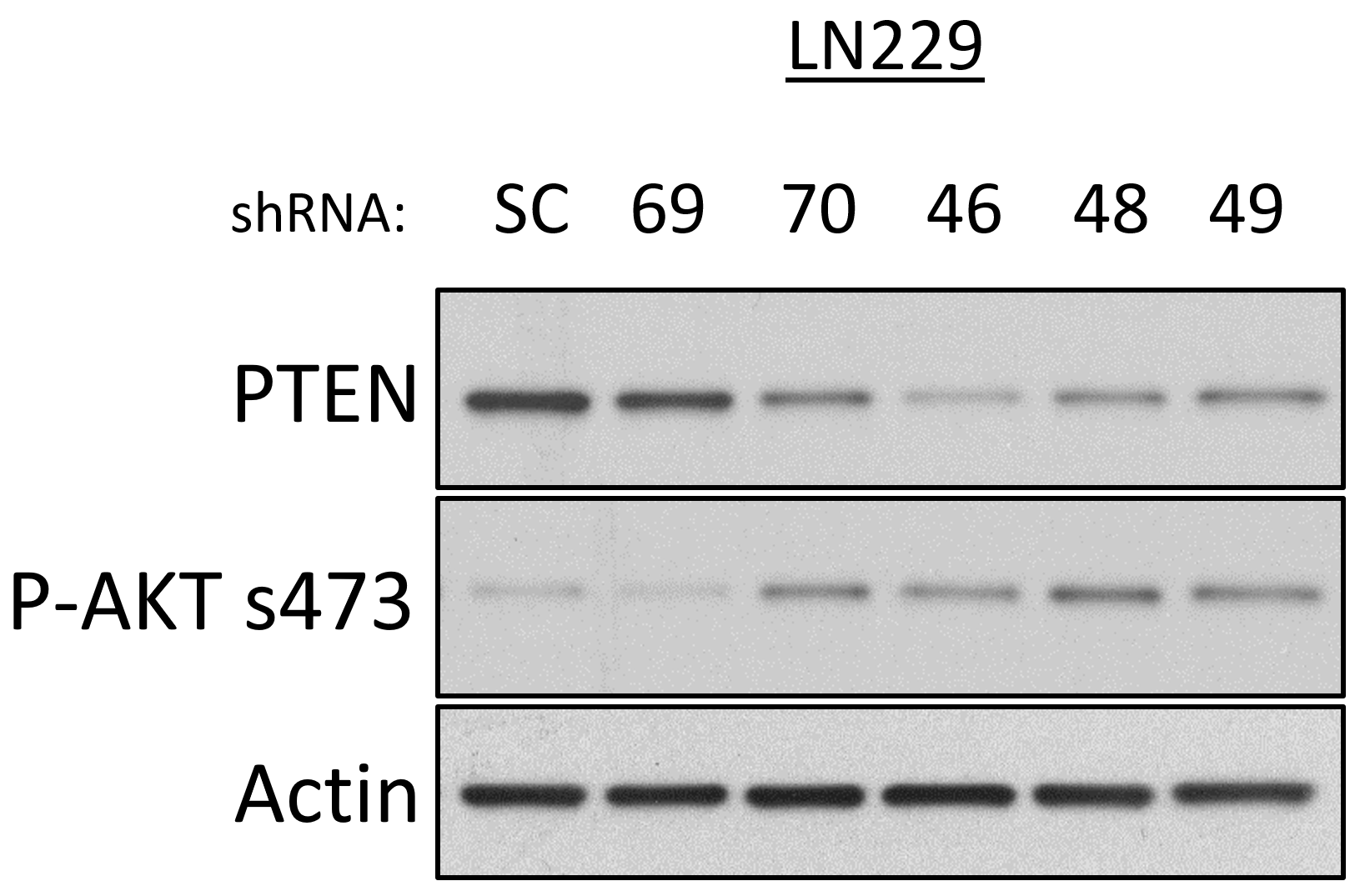
**


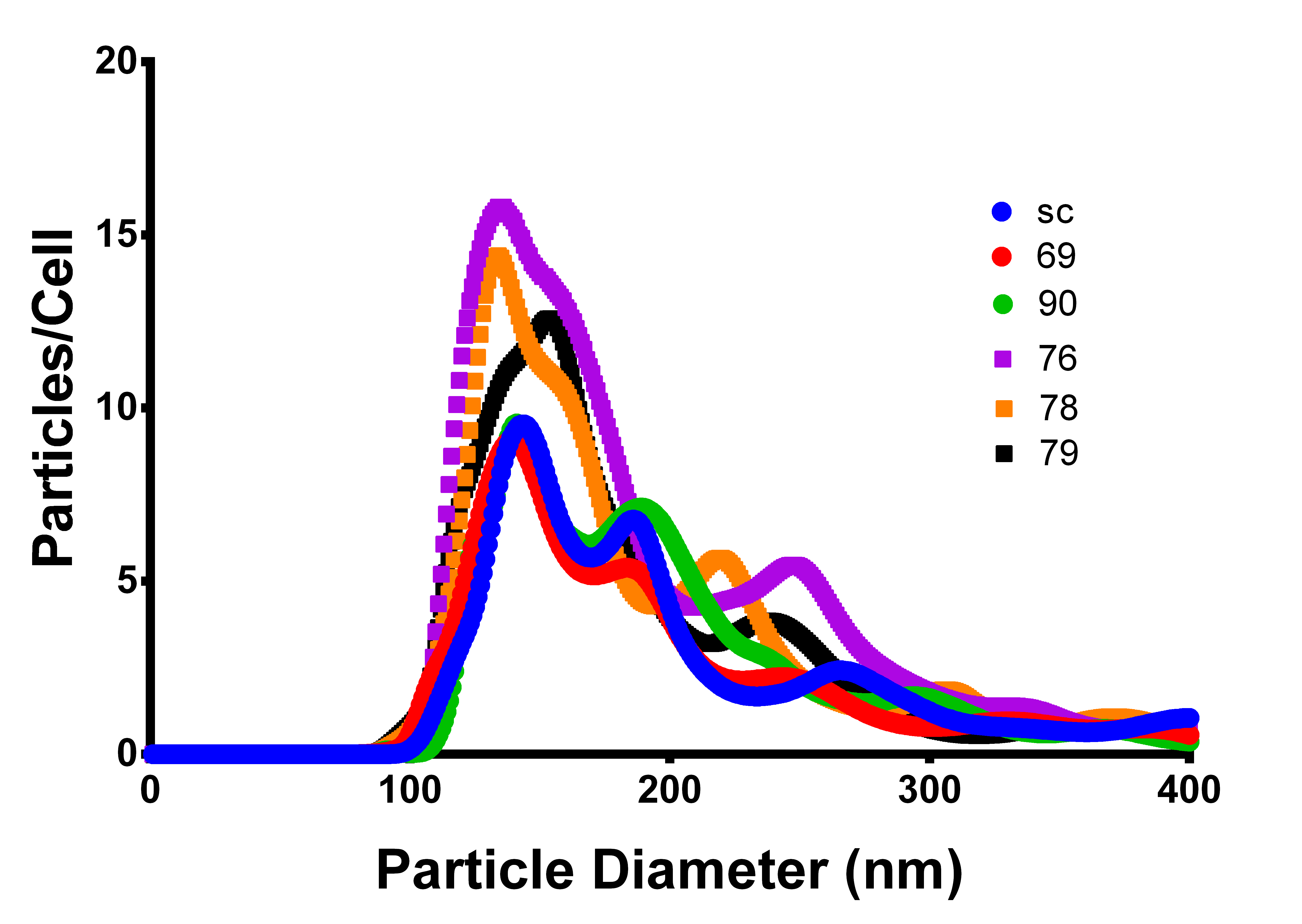


SC
69
70
46
48
49


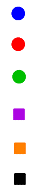


**Supporting Figure 2. KD of PTEN in LN229 cells leads to increased EV biogenesis and PD-L1 cargo**

**A.)** Schematic of how cells were cultured and treated for EV collection. Partially clarified media (PCM) was used for EV analysis, and biological experiments, while the P120 and P15 fractions were utilized for immunoblot analysis. **B.)** LN229 cells were virally transduced with one scramble (SC) shRNA and five different shRNAs targeting PTEN. Each shRNA is represented by the last two digits of Sigma’s MISSION® TRC shRNA numerical identifier as referenced in the “Materials and Methods” section. After 48 hours, cells were washed 3x with PBS or base media and then cultured for 12-15 hours in serum free media conditions. The supernatant was collected, and cells were washed, lysed, and subjected to immunoblot analysis to compare PTEN expression, total AKT, and p-AKT (S473), with actin as the loading control (top). The conditioned media was processed by differential centrifugation to produce PCM and subjected to NTA (bottom). **C)** LN229 cells were transduced with PTEN shRNA 2746 or scrambled shRNA. Forty-eight hours later, cells were treated with IFN-γ (50 ng/mL) for 24 hours and prepared for EV collection as before. Cells were lysed and subjected to immunoblot analysis using antibodies against PD-L1, EGFR, PTEN, AKT, and p-AKT (S473). Actin served as a loading control **D.)** The P120 EV fraction (exosome) was subjected to immunoblot analysis for changes in PD-L1 and EGFR, while CD81 and Flotillin-II served as loading controls. (Left) blot loaded for equal protein content vs. (right) a blot loaded for relative EV output.

**A B**


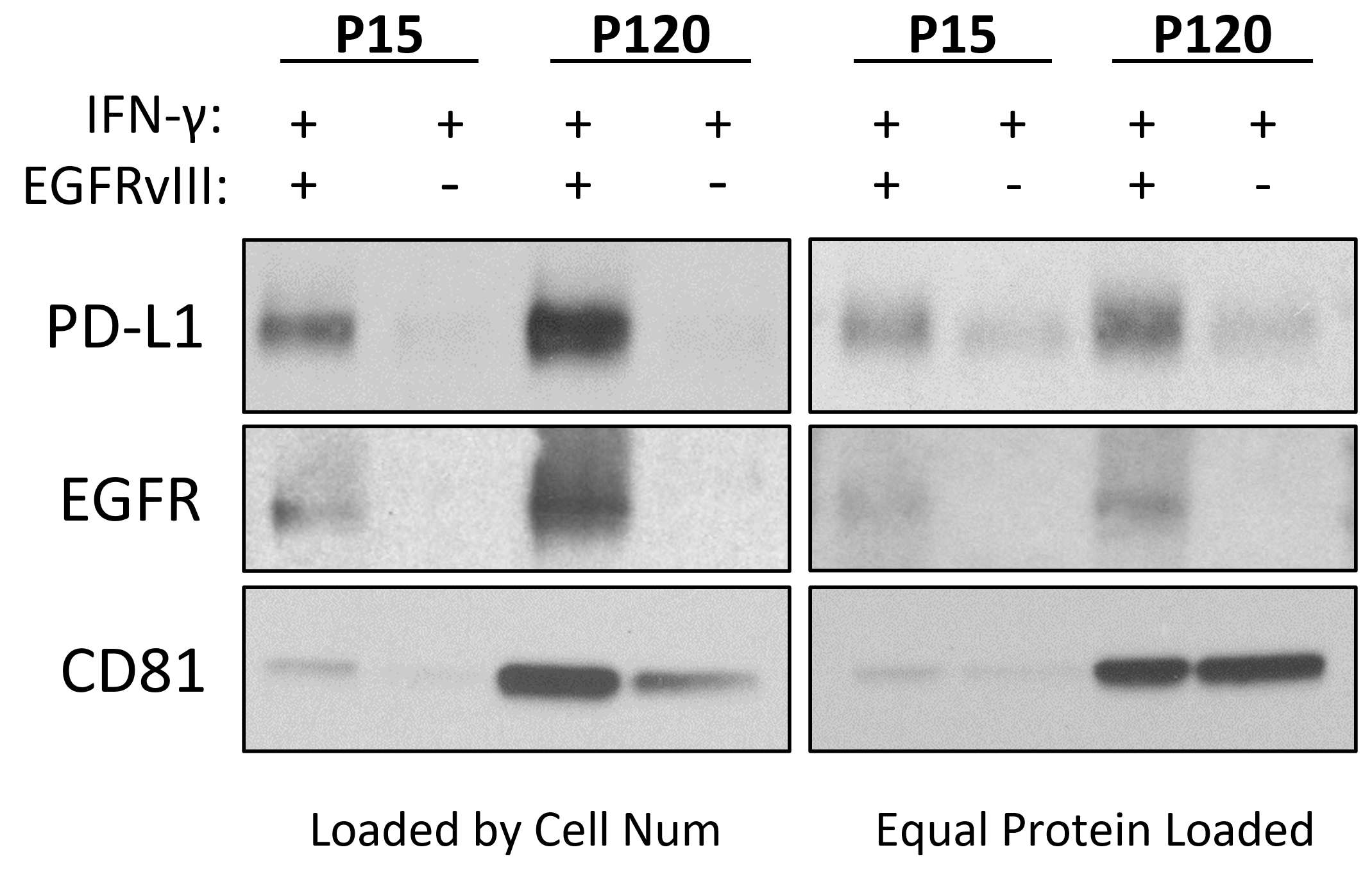

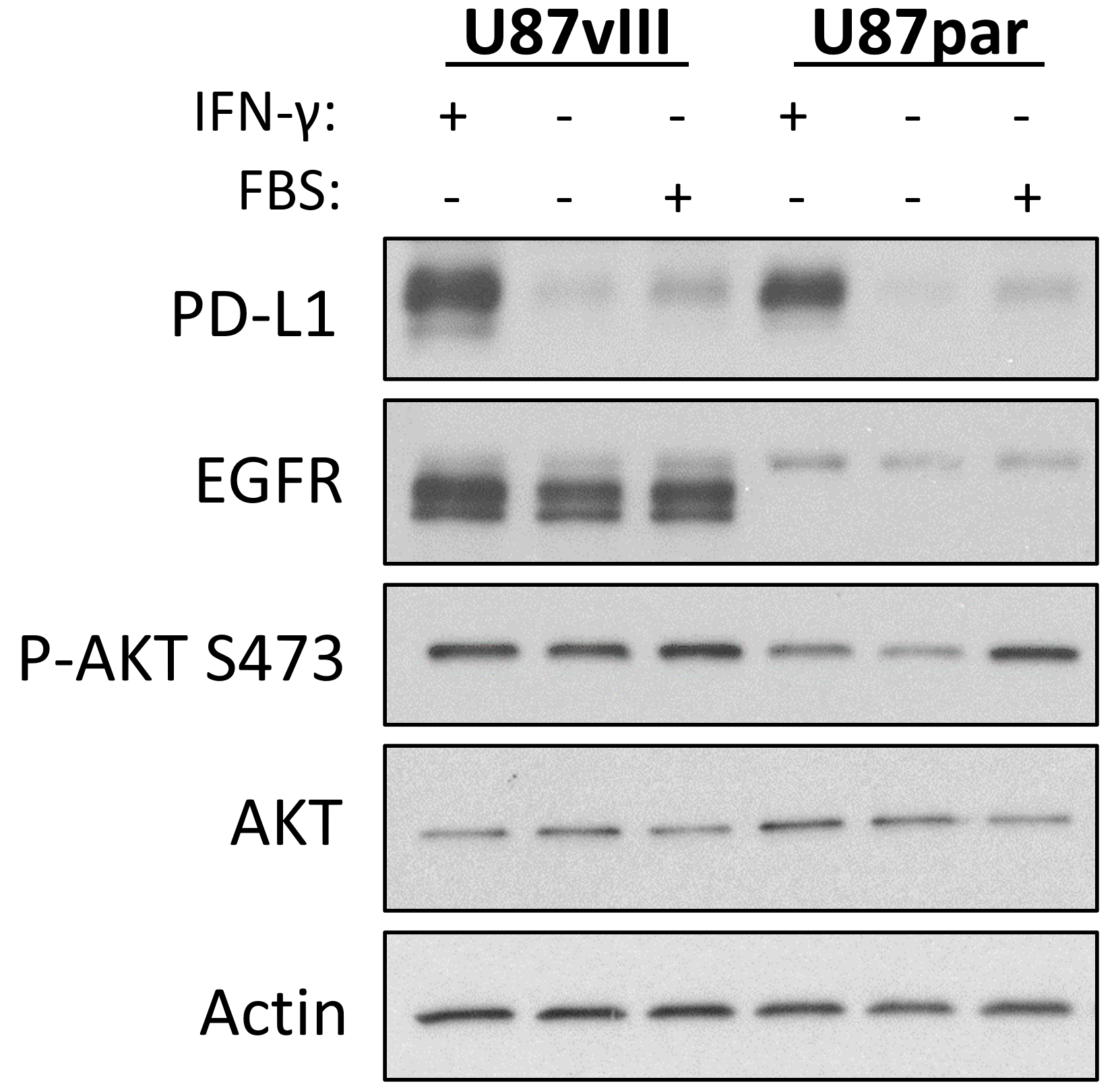


**C**


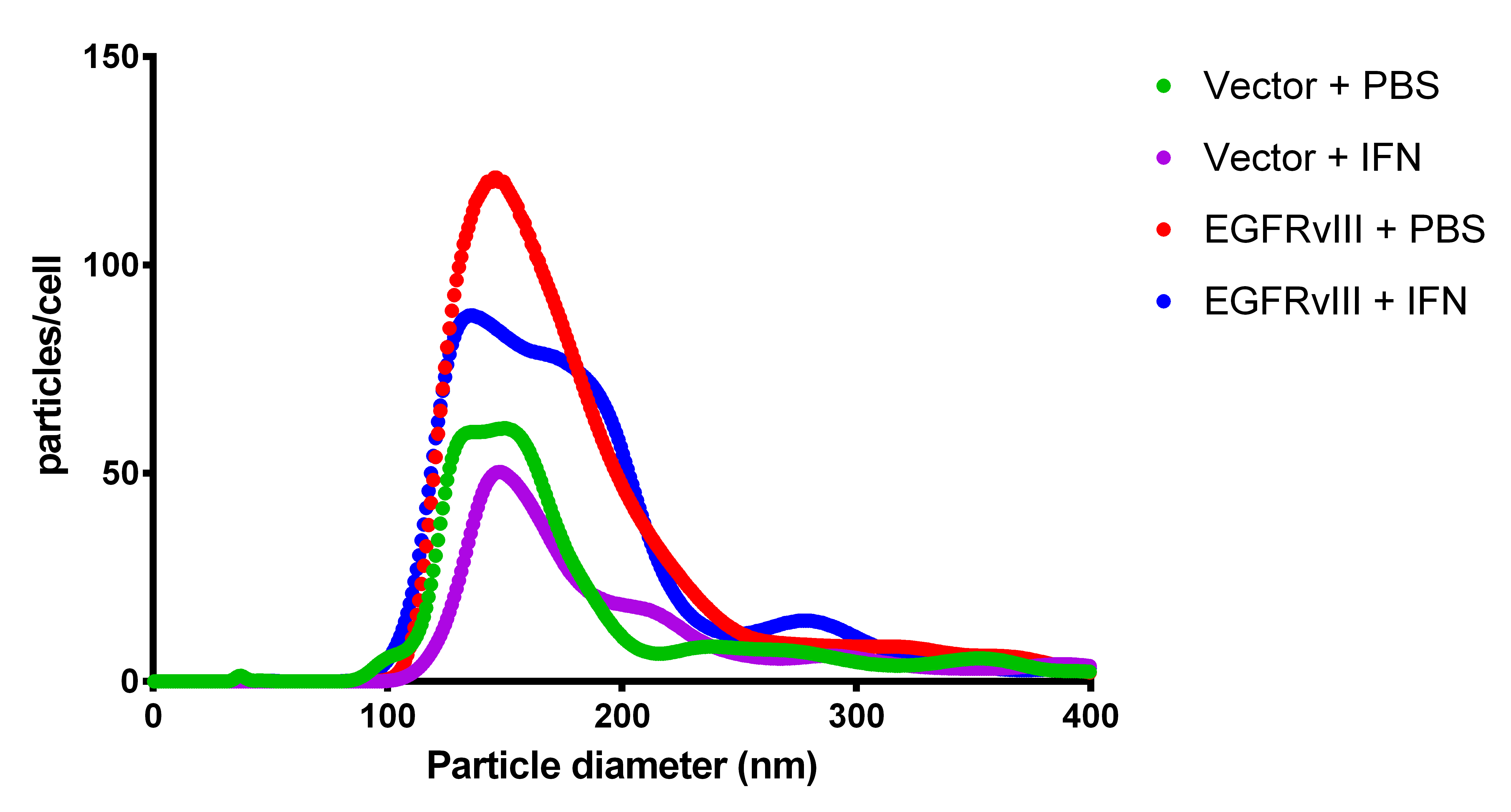

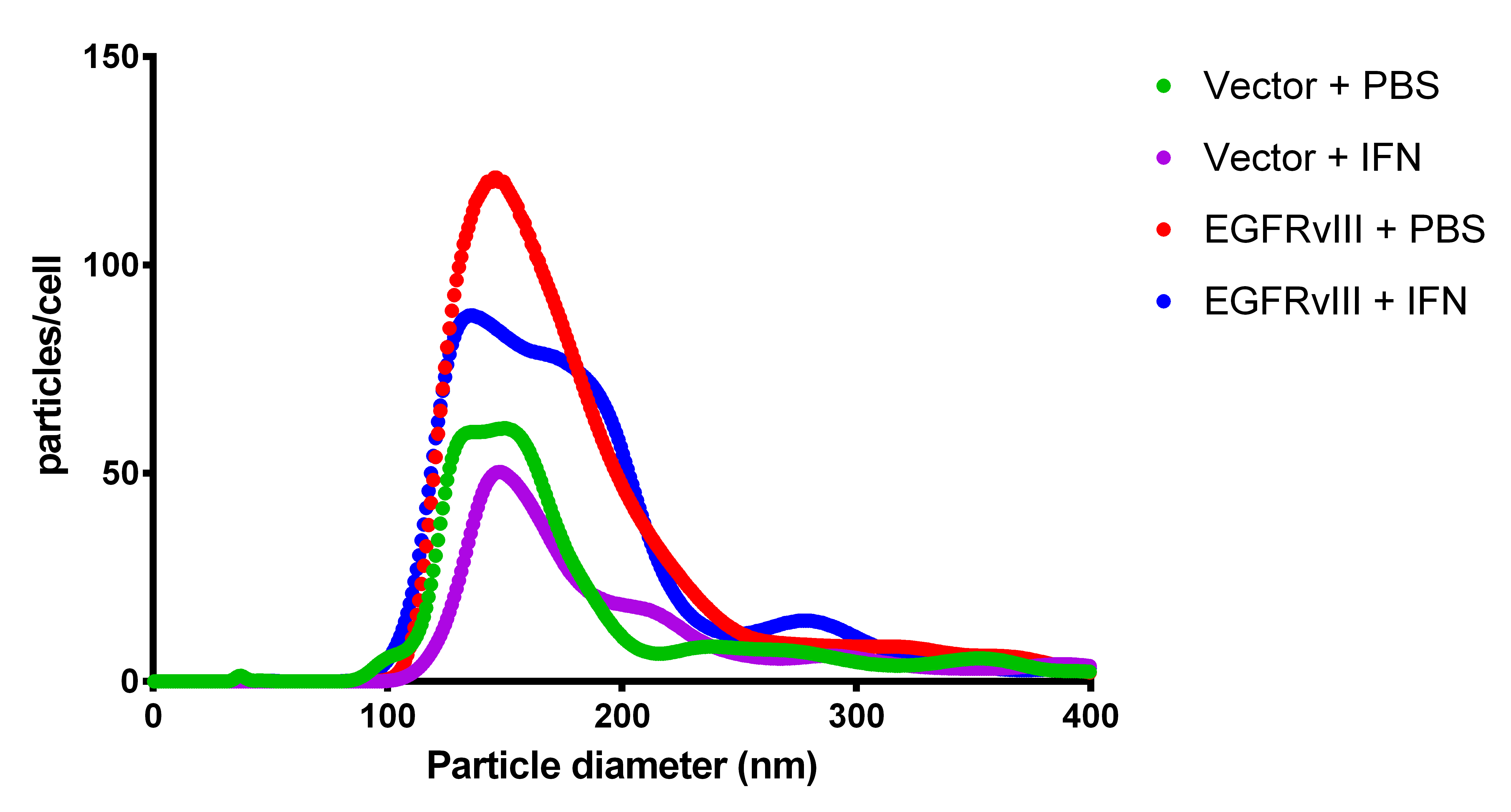

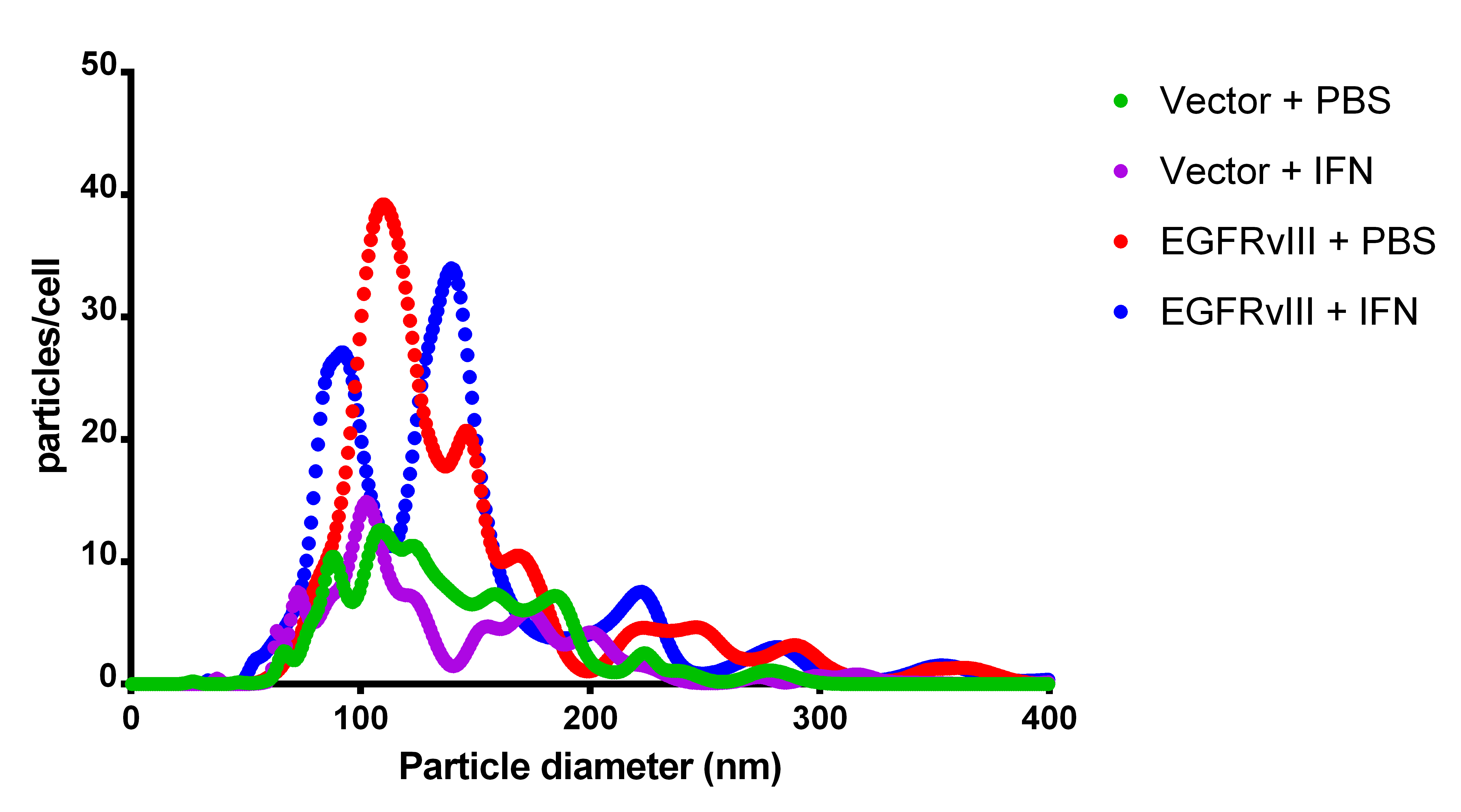

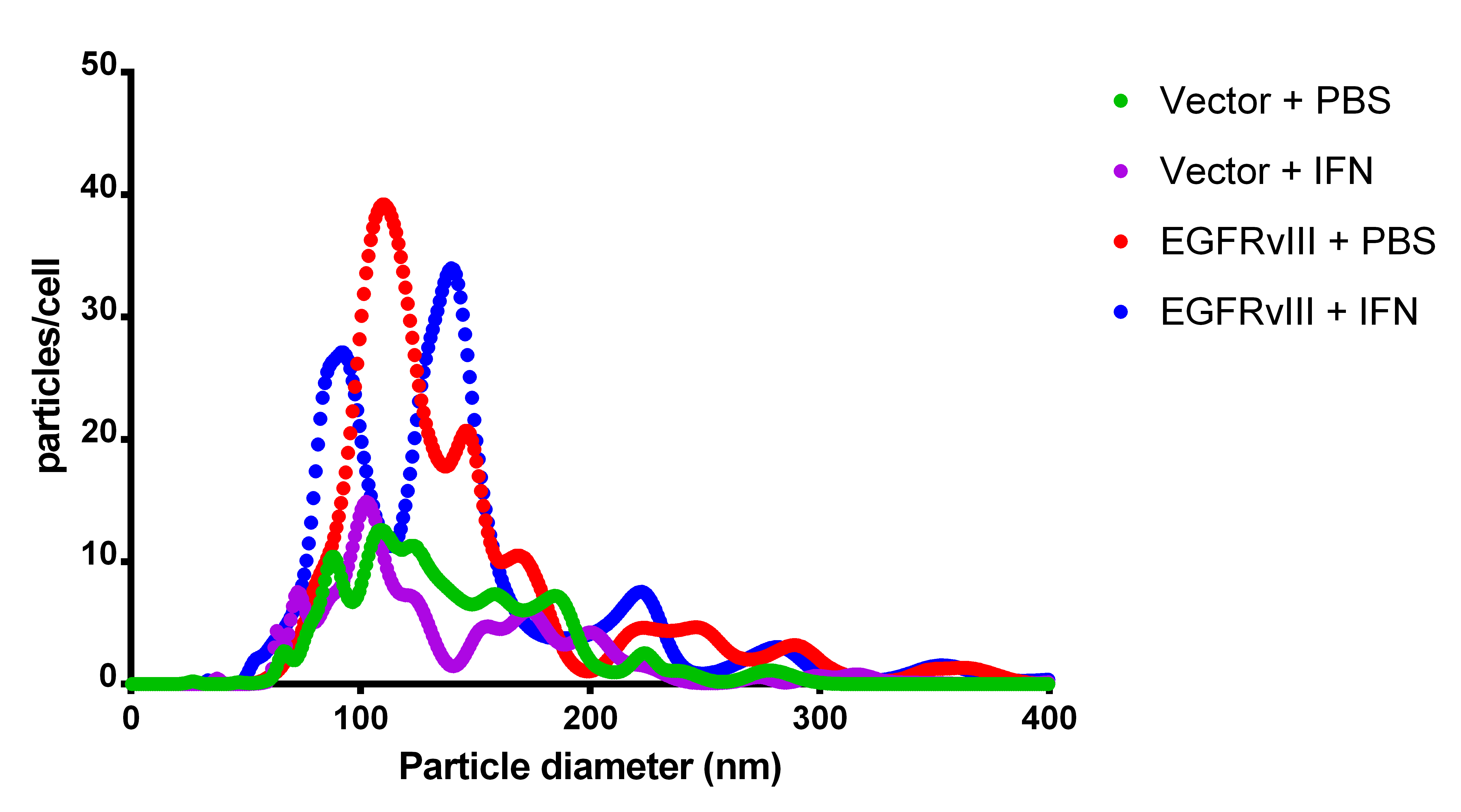

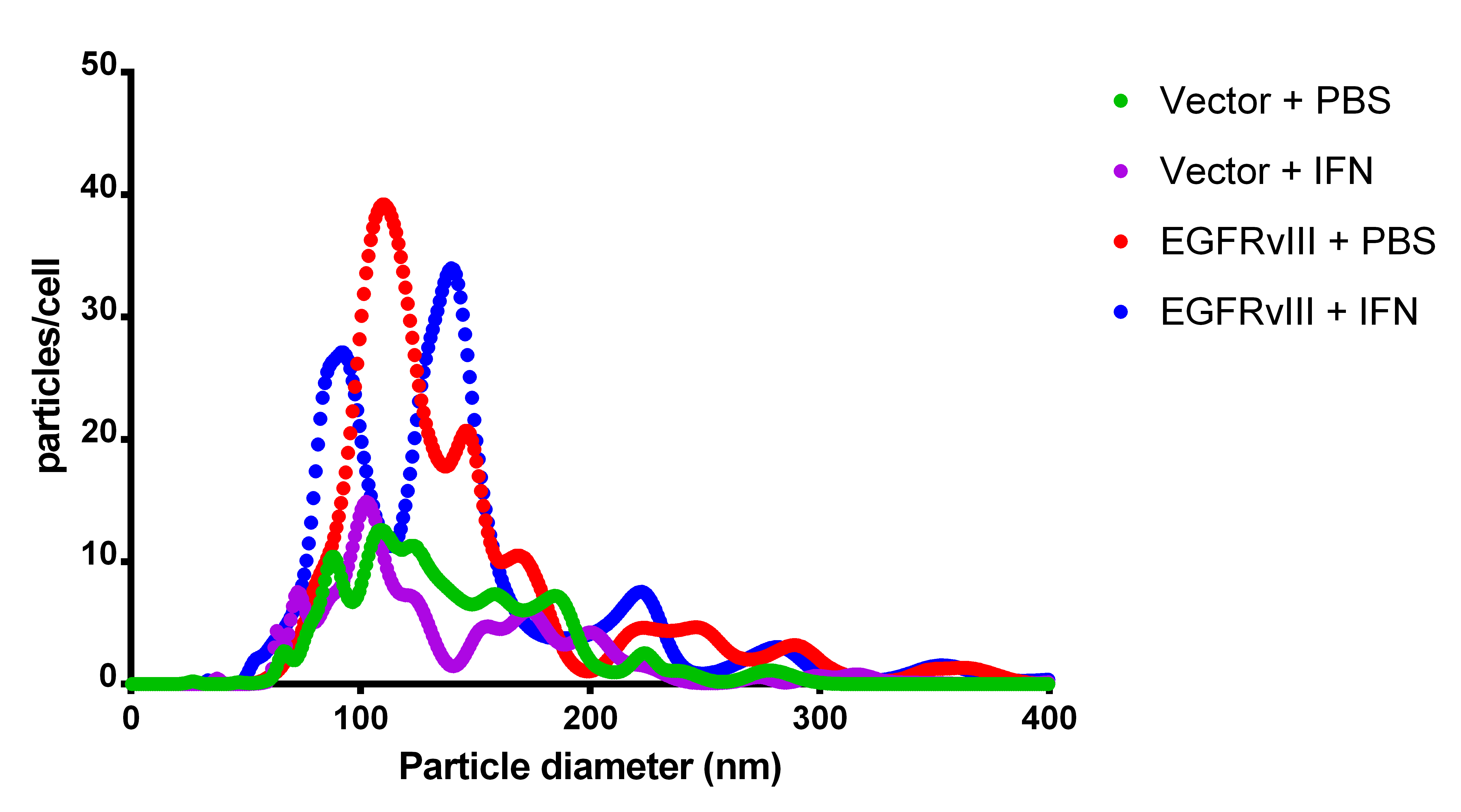

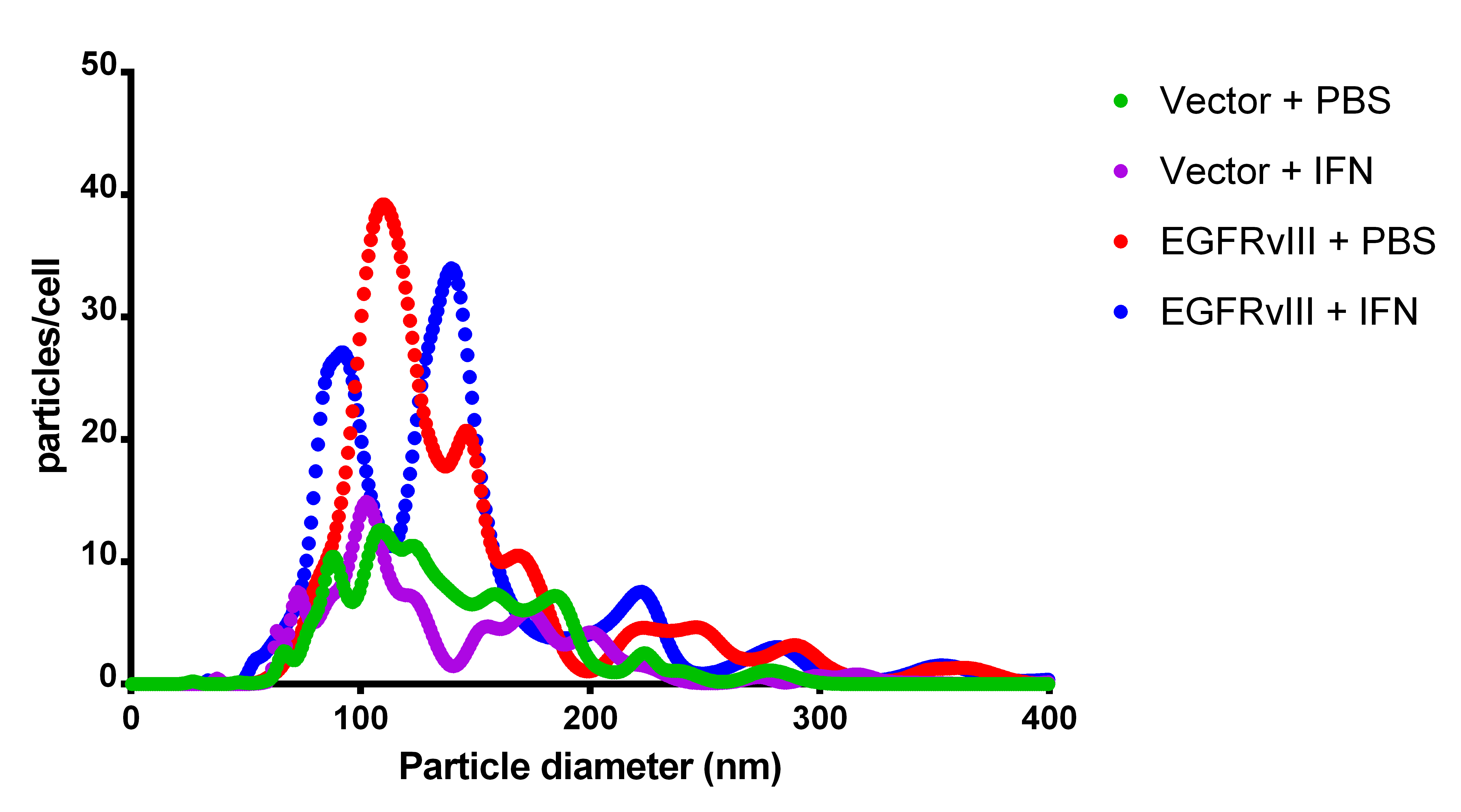


**Supporting Figure 3: Expression of EGFRvIII in U87 cells leads to an increase in EV biogenesis and PD-L1 associated cargo**

**A)** Semi-stable U87 cells expressing EGFRvIII, or empty vector (parental), were treated with or without IFN-γ (50 ng/mL) for 24 hours followed by 12-16 hours in serum free conditions. A separate group of was maintained in complete media containing serum (FBS) without IFN-γ. Cells were then subjected to immunoblot analysis to compare expression of PD-L1 EGFR, AKT, and p-AKT (S473), with actin serving as the loading control. **B)** The PCM from the serum deprived cells was processed as before and analyzed by NTA. **C)** The PCM was further processed by differential centrifugation to isolate the larger P15 EV fraction and smaller P120 EV fraction and analyzed by immunoblot analysis for PD-L1, EGFR, and Ras with CD81 serving as loading marker. On the left, EV lysates were loaded based on cell counts. On the right, a second SDS-PAGE was performed in which EVs were loaded based on equal protein content.


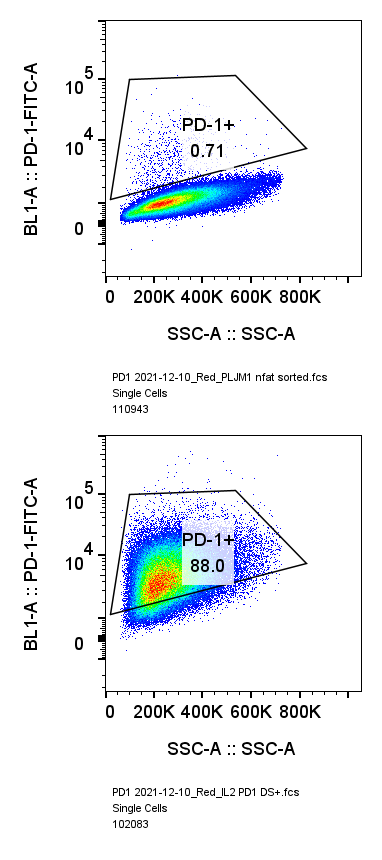

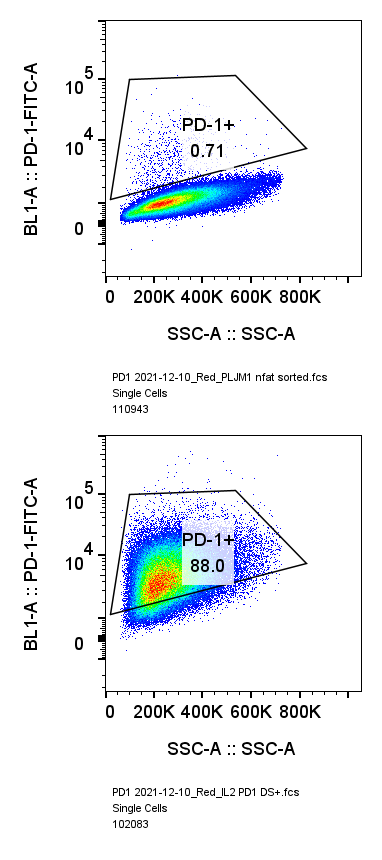
**A B**

**
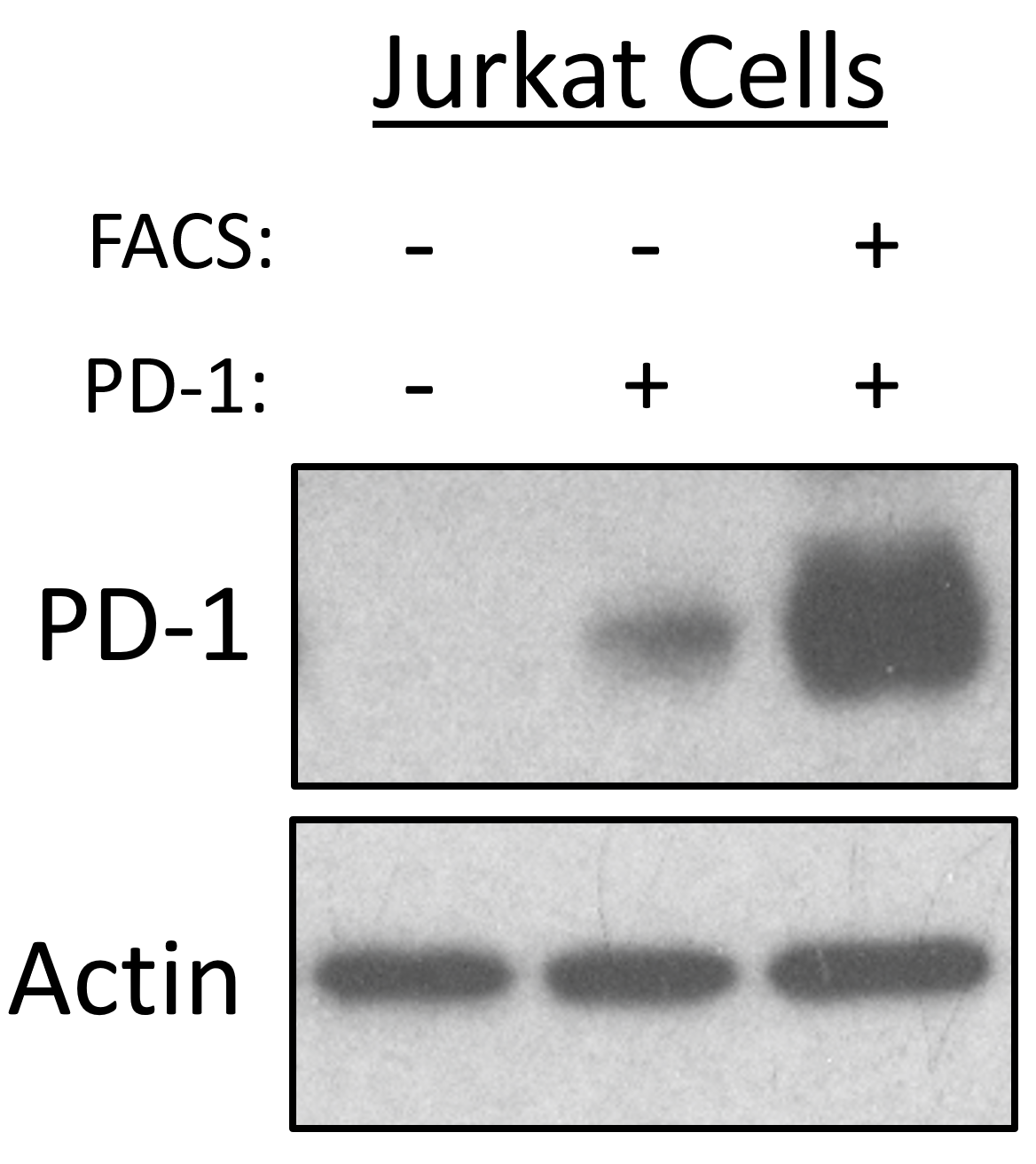
**



**C**





**Supplemental Figure 4: Jurkat NFAT luciferase reporter cells transduced with PD-1 respond to simulating αCD3 antibody**

**A)** The Jurkat T lymphoma cell line expressing the NFAT luciferase reporter was transduced with pLJM1-PD-1 or pLJM1-empty construct at a 1:2 ratio. Three days post-transduction, cells were analyzed by immunoblot for PD-1 expression with actin serving as a loading control. PD-1 transduced Jurkat cells (lane 2) were enriched as described in B) and shown here (lane 3). **B)** Transduced Jurkat cells were sorted by FACS to enrich for high expressors of PD-1. Left, scatter plot showing PD-1 expression on control transduced Jurkat cells compared to the PD-1 transduced and sorted population, right. **C)** Left, sorted Jurkat^PD-1^ cells were treated with increasing concentrations of stimulating αCD3 antibody (OKT3) with three end points of 3-, 6- and 20-hours post-treatment to determine an appropriate dose/time for functional assays. Right, an EC_50_ value for αCD3 antibody (50 ng/mL) was determined and used at the 6-hour time point for the remainder of the assays.
